# Effect of the Y chromosome on plasma high-density lipoprotein-cholesterol levels in Y-chromosome-consomic mouse strains

**DOI:** 10.1186/1756-0500-7-393

**Published:** 2014-06-25

**Authors:** Jun-ichi Suto, Kunio Satou

**Affiliations:** 1 Agrogenomics Research Center, National Institute of Agrobiological Sciences, Tsukuba, Ibaraki 305-8634, Japan; 2 Center for Animal Disease Control and Prevention, National Institute of Animal Health, Tsukuba, Ibaraki 305-0856, Japan

**Keywords:** HDL-cholesterol, *Sry*, *Uty*, Y-chromosome-consomic mouse strains

## Abstract

**Background:**

Plasma high-density lipoprotein (HDL)-cholesterol level is a clinically important quantitative phenotype that widely varies among inbred mouse strains. Several genes or loci associated with plasma HDL-cholesterol levels have been identified on autosomes and the X chromosome. In contrast, genes or loci on the Y chromosome have not attracted significant attention hitherto. Therefore, we investigated the effects of the Y chromosome on plasma HDL-cholesterol levels in Y- chromosome-consomic (Y-consomic) mouse strains.

**Findings:**

Plasma HDL-cholesterol level data from 16 Y-consomic strains demonstrated that the Y chromosome substitutions significantly altered plasma HDL-cholesterol levels, i.e., variations in the plasma HDL-cholesterol level could be partially explained by Y chromosome genes. We obtained the following results from the genotype data on 30 single nucleotide polymorphisms (SNPs), including nonsynonymous and synonymous SNPs and 9 polymorphisms in *Sry*: (1) Variation in rs46947134 of *Uty* was significantly associated with plasma HDL-cholesterol levels. (2) A CAG repeat number polymorphism in *Sry* was significantly associated with plasma HDL-cholesterol levels. (3) Strains with a certain haplotype of the *Mus musculus domesticus*-type Y chromosome had significantly lower plasma HDL-cholesterol levels than strains with a certain haplotype of the *M. m. musculus*-type Y chromosome.

**Conclusions:**

The effect of the Y chromosome on plasma HDL-cholesterol levels was confirmed in the Y-consomic strains. We identified several variants associated with plasma HDL-cholesterol levels. Because the physiological significance of various Y-linked genes remains unclear, the results of this study will provide further insights into the functions of Y-linked genes in lipid metabolism.

## Findings

### Background

Plasma high-density lipoprotein (HDL)-cholesterol level is a clinically important quantitative phenotype that widely varies among inbred mouse strains [1]. Plasma HDL-cholesterol levels are genetically determined to a great extent [2]. The genetic basis of plasma HDL-cholesterol levels have been explored by the means of quantitative trait locus (QTL) mapping, and numerous relevant QTLs have been identified on mouse chromosomes [2]. In addition, the analysis of a series of mouse mutants has provided direct evidence for the presence of plasma HDL-cholesterol genes (MGI, Mouse Genome Informatics; http://www.informatics.jax.org). Most of these genes or loci are autosomal or X-linked, and the Y chromosome has not attracted significant attention hitherto. However, the effect of the Y chromosome on cholesterol levels has been implicated in several human studies, although the findings have been rather controversial [3-9]. To address this controversy, we performed genetic analyses of plasma HDL-cholesterol levels in Y-chromosome-consomic (referred to as Y-consomic hereafter) strains, in which the Y chromosome from various inbred mouse strains had been introgressed onto an inbred DH/Sgn strain background [10,11]. We can eliminate the phenotypic effects of autosomes and the X chromosome using the Y-consomic strains. Because our results suggested that the Y chromosome had significant effects on plasma HDL-cholesterol levels, we also aimed to identify specific associations between plasma lipid level and Y-linked gene polymorphisms. The identification of Y-linked genes associated with plasma HDL-cholesterol levels will provide further insights into the functions of Y-linked genes in lipid metabolism and the inheritance of coronary artery diseases in men [9].

### Methods

The inbred mouse strain DH/Sgn (referred to as DH hereafter) was maintained at the National Institute of Agrobiological Sciences (NIAS, Tsukuba, Japan). We had previously established the following Y-consomic strains: DH-Chr Y^A^ (n = 8), DH-Chr Y^AKR^ (n = 9), DH-Chr Y^B6^ (n = 12), DH-Chr Y^BALB^ (n = 9), DH-Chr Y^C3H^ (n = 12), DH-Chr Y^CAST^ (n = 16), DH-Chr Y^CBA^ (n = 11), DH-Chr Y^CF1^ (n = 8), DH-Chr Y^DDD^ (n = 16), DH-Chr Y^DH^ (identical to DH, n = 11), DH-Chr Y^KK^ (n = 5), DH-Chr Y^RF^ (n = 14), DH-Chr Y^RR^ (n = 16), DH-Chr Y^SJL^ (n = 16), DH-Chr Y^SS^ (n = 10), and DH-Chr Y^SWR^ (n = 9). These Y-consomic strains were also maintained at the NIAS. In total, 182 Y-consomic strain mice were used in this study.

All mice were maintained in a specific pathogen-free facility with a regular 12-h light:12-h dark light cycle, controlled temperature, and humidity. Food (CRF-1, Oriental Yeast Co. Ltd., Tokyo, Japan) and water were freely available throughout the experimental period. All animal procedures were approved by the Institutional Animal Care and Use Committee of NIAS, and the experiments were performed in accordance with the committee-approved guidelines.

At 80 days of age, mice were killed with an overdose of ether after a 4-h fast. Blood was then drawn from the heart into microtubes with heparin as an anticoagulant. The tubes were centrifuged at 7,000 rpm for 5 min at 4°C to separate plasma from whole blood. Plasma samples were maintained at −70°C until use. Plasma HDL-cholesterol levels were enzymatically determined using a spectrophotometer with clinical chemical kits (Test Wako, Wako Pure Chemical Industries Ltd., Osaka, Japan). Plasma HDL-cholesterol levels were determined in sample from which low-density lipoprotein and very low-density lipoprotein fractions had been previously precipitated with phosphotungstic acid and magnesium chloride.

Single nucleotide polymorphism (SNP) genotyping was performed by direct sequencing of the PCR product of the genomic region containing the SNP site. Thirty SNPs were identified in the 15 Y-consomic strains (DH-Chr Y^DH^ was not genotyped). These SNP loci were selected on the basis of SNP data retrieved from the Mouse Phenome Database (MPD, http://phenome.jax.org). A high-density strain set comprising 18 inbred strains had 18 SNPs associated with nonsynonymous amino acid changes. Of them, rs51394161, which was located on exon 5 of *Zfy2*, could not be determined; therefore, 17 nonsynonymous SNPs were genotyped. The MPD search also yielded 25 synonymous SNPs, and of them, 13 were genotyped. In this study, the following SNPs were genotyped: rs47359684, rs47900677, rs46080695, rs52139814, rs45850354, rs48064925, rs51995337, rs51133250, rs50647790, rs51277152, rs48685451, rs48834187, rs46947134, rs51756947, rs48554025, rs47574660, rs51766109, rs49468864, rs49623242, rs51230091, rs49614307, rs48926479, rs51025923, rs48512209, rs47293184, rs51685350, rs47616691, rs51560704, rs46643293, and rs51529727.

The nucleotide (nt) sequence of *Sry* was also determined by direct sequencing of the PCR product. *Sry* polymorphisms included nucleotide substitutions at seven sites and a number of major CAG repeats at two sites [12]. The following polymorphisms were genotyped: nt 8491, nt 8701, nt 8711, nt 8731, number of first CAG repeats starting at nt 8733, number of second CAG repeats starting at nt 8811, nt 8930, nt 8934, and nt 9006. The nt numbers are based on the GenBank entry X67204.

Trait data distribution normality in Y-consomic mice was tested using the Shapiro-Wilk W test (JMP8, SAS Institute Japan Inc., Tokyo).

Y-linked genetic variations controlling plasma HDL-cholesterol levels were identified using the following three-step approach [11]: (1) The effects of genes on autosomes and the X chromosome were eliminated by using Y-consomic strains, and the net phenotypic effects of Y-linked genes were assessed. (2) The Dunnett’s multiple comparison test or Steel test, with the background DH strain as a reference, was used to determine if a trait was Y-linked. (3) The data from all strains were assembled on the basis of SNP genotypes and the statistical significance of differences was assessed. Two groups partitioned by genotype were compared using the Student’s or Welch’s *t*-test, and three groups were compared with one-way analysis of variance (ANOVA). On the basis of the number of SNP loci (n) genotyped, the significant threshold *P* value was determined as 0.05/n with the Bonferroni correction test.

Statistical comparisons among the haplotype-based Y-consomic strain groups were performed with Tukey–Kramer honest significant difference (HSD) tests.

### Results and discussion

Among the Y-consomic strains, the DH-Chr Y^A^, DH-Chr Y^B6^, DH-Chr Y^BALB^, DH-Chr Y^C3H^, DH-Chr Y^CBA^, DH-Chr Y^CF1^, DH-Chr Y^DH^, DH-Chr Y^KK^, DH-Chr Y^RR^, and DH-Chr Y^SS^ strains possess the *Mus musculus musculus*-type Y chromosome (Y^Mus^), whereas the DH-Chr Y^AKR^, DH-Chr Y^DDD^, DH-Chr Y^RF^, DH-Chr Y^SJL^, and DH-Chr Y^SWR^ strains possess the *M. m. domesticus*-type Y chromosome (Y^Dom^). The strains, Y^Mus^ vs. Y^Dom^, were classified on the basis of the following criteria: (1) a C-to-T transitional substitution at nt 8491 in the high mobility group (HMG) box of *Sry* (Y^Mus^ had T and Y^Dom^ had C) [13] and (2) the presence of a C-to-T change that created a TAG termination codon at nt 9006 in the third major CAG repeat starting at nt 8985 in Y^Dom^[14], which was absent in Y^Mus^ (the mouse *Sry* gene has four major sites consisting approximately 10 CAG repeats). The partitioning of the strains into either Y^Mus^ or Y^Dom^ was compatible with the descriptions in the previous studies [15,16].

Plasma HDL-cholesterol levels in 182 mice from 16 Y-consomic strains exhibited a bell-shaped distribution curve but did not strictly follow a normal distribution (data not shown). However, when the data from the DH-Chr Y^DH^ strain was excluded (i.e., 171 mice from 15 strains), the plasma HDL-cholesterol level values followed a normal distribution. The DH-Chr Y^DH^ strain was excluded because SNP/*Sry* genotyping was not performed in this strain, and it is not included in the subsequent statistical analyses.

The plasma HDL-cholesterol level was compared for each Y-consomic strain (Table 1). According to the Steel test results, the DH-Chr Y^CF1^, DH-Chr Y^DDD^, DH-Chr Y^B6^, DH-Chr Y^SJL^, DH-Chr Y^AKR^, DH-Chr Y^SWR^, and DH-Chr Y^KK^ strains exhibited significantly lower plasma HDL-cholesterol levels than the DH-Chr Y^DH^ strain. Therefore, the Y chromosome substitution had a significant influence on plasma HDL-cholesterol levels.

**Table 1:** Plasma high-density lipoprotein (HDL)-cholesterol levels in Y-consomic strains

**Y chromosome donor strain**	**Plasma HDL-cholesterol level (mg/dl, mean ± SE)**	**P value vs. DH**	**Sample size**
DH	102.4 ± 2.1		11
A	100.2 ± 2.0	0.9952	8
C3H	98.6 ± 2.8	0.9965	12
SS	97.2 ± 4.1	0.9999	10
CBA	96.2 ± 2.5	0.5958	11
CAST	94.7 ± 3.3	0.8287	16
RR	93.2 ± 2.4	0.0683	16
RF	93.0 ± 2.3	0.1198	14
BALB	89.5 ± 3.4	0.0790	9
CF1	89.1 ± 1.8	0.0172	8
DDD	85.7 ± 2.3	0.0037	16
B6	84.7 ± 1.8	0.0018	12
SJL	84.3 ± 1.8	0.0011	16
AKR	83.8 ± 2.4	0.0046	9
SWR	82.5 ± 4.8	0.0131	9
KK	76.7 ± 3.5	0.0254	5

Table 2 shows the typical polymorphic patterns among 30 SNPs and 9 *Sry* polymorphisms identified in the 15 Y-consomic strains [11]. Most SNPs/polymorphisms could be classified as one of these patterns. These SNP loci were selected on the basis of SNP data retrieved from MPD. A high-density strain set, comprising 18 inbred strains, had 18 SNPs associated with nonsynonymous amino acid changes. Of them, rs51394161, which was located on exon 5 of *Zfy2*, could not be determined; therefore, 17 nonsynonymous SNPs were genotyped. In addition, an MPD search yielded 25 synonymous SNPs, and of them, 13 were genotyped. *Sry* polymorphisms included nt substitutions at seven sites and a number of major CAG repeats at two sites [12].

**Table 2:** **Patterns of rs46947134 and *Sry *** **polymorphism in Y-consomic strains**

**Gene**	**SNP/polymorphism**	**A**	**B6**	**BALB**	**C3H**	**CBA**	**CF1**	**KK**	**RR**	**SS**	**CAST**	**AKR**	**DDD**	**RF**	**SJL**	**SWR**
*Uty*	rs46947134	C	C	C	G	G	C	C	G	C	C	C	C	C	C	C
*Sry*	No. of CAG repeats starting at nt 8733	11	11	11	12	12	11	11	12	11	11	9	9	9	9	9
*Sry*	No. of CAG repeats starting at nt 8811	12	12	12	10	10	12	12	10	12	12	13	12	13	12	12
*Sry*	Nts at 8491 and 8711	T	T	T	T	T	T	T	T	T	C	C	C	C	C	C
*Usp9y*	rs51766109	C	C	C	T	T	C	C	T	C	T	T	T	T	T	T
*Usp9y*	rs49468864^1)^	A	A	A	A	A	A	A	A	A	A	G	G	G	G	G

1) In addition to rs49468864 in *Usp9y*, many SNPs showed similar polymorphic pattern to rs49468864 [11].

Table 3 summarizes the results of the statistical analyses. Mice were divided into two or three groups according to the SNP or polymorphism in their *Sry* gene. Statistically significant differences in mean values between or among groups were then tested. On the basis of the Bonferroni correction test, the significance threshold at α = 0.05 level was 0.00128 because 39 polymorphisms were examined.

**Table 3:** Effects of gene polymorphisms on plasma high-density lipoprotein (HDL)-cholesterol level in Y-consomic strains

**SNP/Gene**	**Polymorphism**	**Plasma HDL-cholesterol level (mg/dl, mean ± SE)**			**P value**
rs46947134		C	G		
(*Uty*)		(n = 132)	(n = 39)		
		88.8 ± 0.9	95.7 ± 1.5		0.00046
*Sry*	No. of CAG repeats starting at nt 8733	9	11	12	
		(n = 64)	(n = 68)	(n = 39)	
		86.2 ± 1.2	91.3 ± 1.4	95.7 ± 1.5	4.73 × 10^–5^
*Sry*	No. of CAG repeats starting at nt 8811	10	12	13	
		(n = 39)	(n = 109)	(n = 23)	
		95.7 ± 1.5	88.7 ± 1.1	89.4 ± 1.9	NS (0.0021)^2)^
*Sry*	Nts at nt 8491 and 8711	T	C		
		(n = 91)	(n = 80)		
		92.6 ± 1.1	87.9 ± 1.2		NS (0.0050)
rs51766109		C	T		
(*Usp9y*)		(n = 52)	(n = 119)		
		90.2 ± 1.5	90.5 ± 1.0		NS (0.90)
rs49468864^1)^		A	G		
(*Usp9y*)		(n = 107)	(n = 64)		
		92.9 ± 1.1	86.2 ± 1.2		5.27 × 10^–5^

1) In addition to rs49468864 in *Usp9y*, many SNPs showed a similar polymorphic pattern to rs49468864 [11].

2) NS: not significant.

In the analysis of variation in the gene for the ubiquitously transcribed tetratricopeptide repeat gene, Y chromosome (*Uty*; rs46947134) was significantly associated with the plasma HDL-cholesterol level. Strains with a C allele had significantly lower plasma HDL-cholesterol levels than those with a G allele. rs46947134 was a nonsynonymous SNP and was accompanied by a His-to-Asp amino acid change, but the physiologic significance of this amino acid change remains unclear. In humans, Russo et al. [8] explored genetic variants and reported the absence of a polymorphism in *UTY* in three ethnic groups. Recently, Bloomer et al. [17] observed that an increased risk of coronary artery disease was associated with reduced *UTY* expression. However, it is unknown whether the G/C variants in mouse *Uty* are associated with the expression level.

The number of first major CAG repeats, starting at nt 8733 of *Sry*, was significantly associated with plasma HDL-cholesterol levels. Strains with 9 CAG repeats had significantly lower plasma HDL-cholesterol levels than those with 11 or 12 repeats. Because *Sry* sequences, except those for HMG boxes, are poorly conserved among species, and *Sry* genes other than mouse do not possess a CAG-repeat stretch, it is impossible to directly apply the results from mouse studies to other mammalian species [18]. Nevertheless, *Sry* has been recently described to play roles other than testis determination [19]. An association between the Y chromosome and blood pressure has been repeatedly reported in rats and humans [3,20-24], and *Sry* is thought to be the most promising candidate for the Y chromosomal effect [23]. Because both hypertension and dyslipidemia are major components in determining cardiovascular risk, it is not surprising that *Sry* plays role in lipid metabolism. Overall, we found 22 polymorphic SNPs, as represented by rs49468864 in *Usp9y*, were significantly associated with plasma HDL-cholesterol levels.

On the basis of the six SNPs and *Sry* polymorphisms haplotypes (Table 2), Y-consomic strains were partitioned into five groups (Table 4). Tukey–Kramer HSD tests were used to compare plasma HDL-cholesterol levels among the five haplotype-based groups. A significant difference was observed between Groups 1 and 5 (P < 0.0001). Group 1 consisted of strains with Y^Dom^, whereas Group 5 consisted of strains with Y^Mus^. Therefore, it was concluded that strains with a certain haplotype of Y^Dom^ had significantly lower plasma HDL-cholesterol levels than strains with a certain haplotype of Y^Mus^. However, it cannot be said that the differences in HDL-cholesterol levels among the Y-consomic strains were neatly and tidily explained by partitioning based on Y-linked haplotypes.

**Table 4:** **Plasma HDL-cholesterol level in Y-consomic strains partitioned by haplotypes based on Y-linked SNPs and *Sry *** **polymorphisms**

**Group**	**Strains**	**Sample size**	**Plasma HDL-cholesterol level (mg/dl, mean ± SE)**
1	DDD	41	84.4 ± 1.6
	SJL		
	SWR		
2	AKR	23	89.4 ± 2.1
	RF		
3	A	52	90.2 ± 1.4
	B6		
	BALB		
	CF1		
	KK		
	SS		
4	CAS	16	94.7 ± 2.6
5	C3H	39	95.7 ± 1.6
	CBA		
	DBA		
	RR		

The effect of the Y chromosome on plasma HDL-cholesterol levels was confirmed in the Y-consomic strains. We identified several variants associated with plasma HDL-cholesterol levels. Because the physiological significance of various Y-linked genes remains unclear, the results of this study will provide further insights into the functions of Y-linked genes in lipid metabolism.

### Conclusion

The effect of the Y chromosome on plasma HDL-cholesterol levels was confirmed in Y-consomic mouse strains. We identified several genetic variants associated with plasma HDL-cholesterol levels. Because the physiological significance of many Y-linked genes remains unclear, the results of this study provide new insights into the functions of Y-linked genes in lipid metabolism.

## Competing interests

The authors declare that they have no competing interests.

## Authors’ contributions

JS and KS conceived the study. JS conducted the experiments on mice. JS and KS performed data analyses. JS and KS wrote the manuscript. Both authors read and approved the final manuscript.
